# An imbalance of netrin‐1 and DCC during nigral degeneration in experimental models and patients with Parkinson's disease

**DOI:** 10.1111/cns.14141

**Published:** 2023-02-27

**Authors:** Ye Hua, Wenjing Han, Linfeng Zhou, Jing Gao, Jifeng Zhao, Nanshan Song, Bin Hu, Qingyu Yao, Yumin Liu, Deen Xu, Yunnan Lu, Yi Fan

**Affiliations:** ^1^ Department of Neurology, Wuxi No.2 People's Hospital Jiangnan University Medical Center Wuxi China; ^2^ Neuroprotective Drug Discovery Center of Nanjing Medical University, Department of Pharmacology Nanjing Medical University Nanjing China; ^3^ Department of Clinical Laboratory, Wuxi No.2 People's Hospital Jiangnan University Medical Center Wuxi China; ^4^ Department of Neurology Xishan People's Hospital of Wuxi City Wuxi China

**Keywords:** DCC, netrin‐1, neurodegeneration, Parkinson's disease

## Abstract

**Aims:**

Multiple guidance cues, such as netrin‐1 (NTN‐1)/deleted in colorectal carcinoma (DCC), control the guidance of axons and help establish functional neural circuits during development. However, the function of these guidance molecules during the neurodegenerative process is unclear.

**Methods:**

To access the alterations of NTN‐1 and DCC during the onset and progression of PD, we first established two subacute and one chronic PD model. Then, we investigated the relationship between the NTN‐1/DCC pathway and cell death in SH‐SY5Y cells. Finally, we conducted correlation studies between plasma NTN‐1 and parkinsonian symptoms in patients to understand how this pathway contributes to PD.

**Results:**

We found that the imbalance of NTN‐1 and DCC was a common feature of nigral DA neuron injury in PD mouse models. We investigated that MPP+ inhibited NTN‐1 expression and increased DCC expression in a concentration‐ and time‐dependent manner. We further discovered a significant decrease in plasma NTN‐1 levels and a positive correlation with UPDRS scores in PD patients.

**Conclusion:**

Our findings confirmed the imbalance of NTN‐1/DCC signaling during nigral degeneration in experimental PD models and found for the first time a correlation of plasma NTN‐1 with PD symptoms in patients.

## Graphical abstract

As a common feature of nigral DA neuron injury in the early stages, the imbalance of NTN‐1 and DCC appears before cell death. Further studies are needed to clarify the potential differences between central and peripheral NTN‐1.

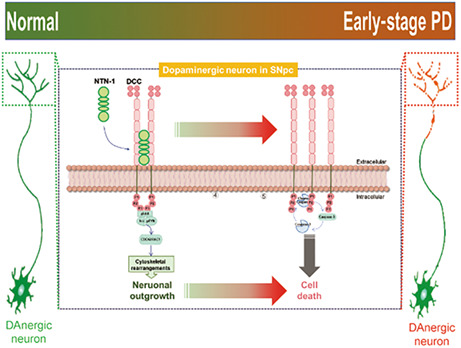

## 1 INTRODUCTION

Human brains contain hundreds of billions of nerve cells that form a complex network of connections and provide the substrate for information processing. Depending on the environment and experience, this neural network can be constantly reconnected and engineered. The imbalances in network signals can cause toxicity, damage, and death of neurons, which then impairs neural communication processes, leading to neurological diseases.^1^, ^2^, ^3^, ^4^, ^5^ Growing evidence suggests that connectivity disruption in neural circuits is a precursor to neuronal death in Parkinson's disease (PD).^1^, ^6^, ^7^ The impairments of mitochondrial dynamics, axonal trafficking, synaptic protein expression, and synaptic integrity are observed before the neurodegeneration in PD.^8^, ^9^, ^10^ Furthermore, disrupted connectivities of neural networks, such as synaptic dysfunction or synaptic loss, are the neuropathological hallmarks of early stage PD.^11^, ^12^ A strategy to reconnect neural circuits might therefore be able to slow the clinical progression of PD.

During development, multiple guidance cues can control the guidance of axons to their specific targets and help establish functional neural circuits, such as netrin‐1 (NTN‐1)/deleted in colorectal carcinoma (DCC), Slit3/robo, Semaphorin5A/plexin, and ephtinB/ephB pathways.^13^ However, the function of these guidance molecules in the adult brain, particularly during the neurodegenerative process, is unclear. Among these, NTN‐1 and its receptor DCC play a critical role in the development and function of the midbrain dopamine circuitry.^14^, ^15^ Some studies have shown that these guidance molecules are significant predictors of PD outcomes,^16^, ^17^ whereas others found that these guidance molecules have a weak association with PD.^18^, ^19^ Recently, some findings provide evidence that the NTN‐1/DCC pathway modulates the survival and death of dopamine neurons and may contribute to non‐motor and motor symptoms in PD.^20^, ^21^ These results confirm a key role of the NTN‐1/DCC pathway in adult dopamine neuron fate in PD. However, it is still unknown how the NTN‐1/DCC pathway is altered during the onset and progression of PD.

In the present study, we first examined changes in the NTN‐1/DCC pathway in multiple animal models of PD and altered NTN‐1/DCC signaling in SH‐SY5Y cells after exposure to 1‐Methyl‐4‐phenyl pyridinium iodide (MPP+). Then, when comparing the plasma level of NTN‐1 between PD patients and healthy controls (HC), we investigated the correlation between plasma levels of NTN‐1 and clinical symptoms of PD.

## 2 METHODS

### 2.1 Animals

Experiments were approved by the Institutional Animal Care and Use Committee of Nanjing Medical University and followed the 3R rules. C57BL/6 mice (male, aged 10–12 weeks, weighing 23–25 g) were purchased from the Animal Core Facility of Nanjing Medical University (Nanjing, Jiangsu, China). All mice were housed in five per cage groups in a standardized light‐dark cycle at 22°C and were fed standard rodent food and water.

### 2.2 Preparation of MPTP mouse model and LPS mouse model

#### 2.2.1 Subacute MPTP model of PD

**FIGURE 1:**
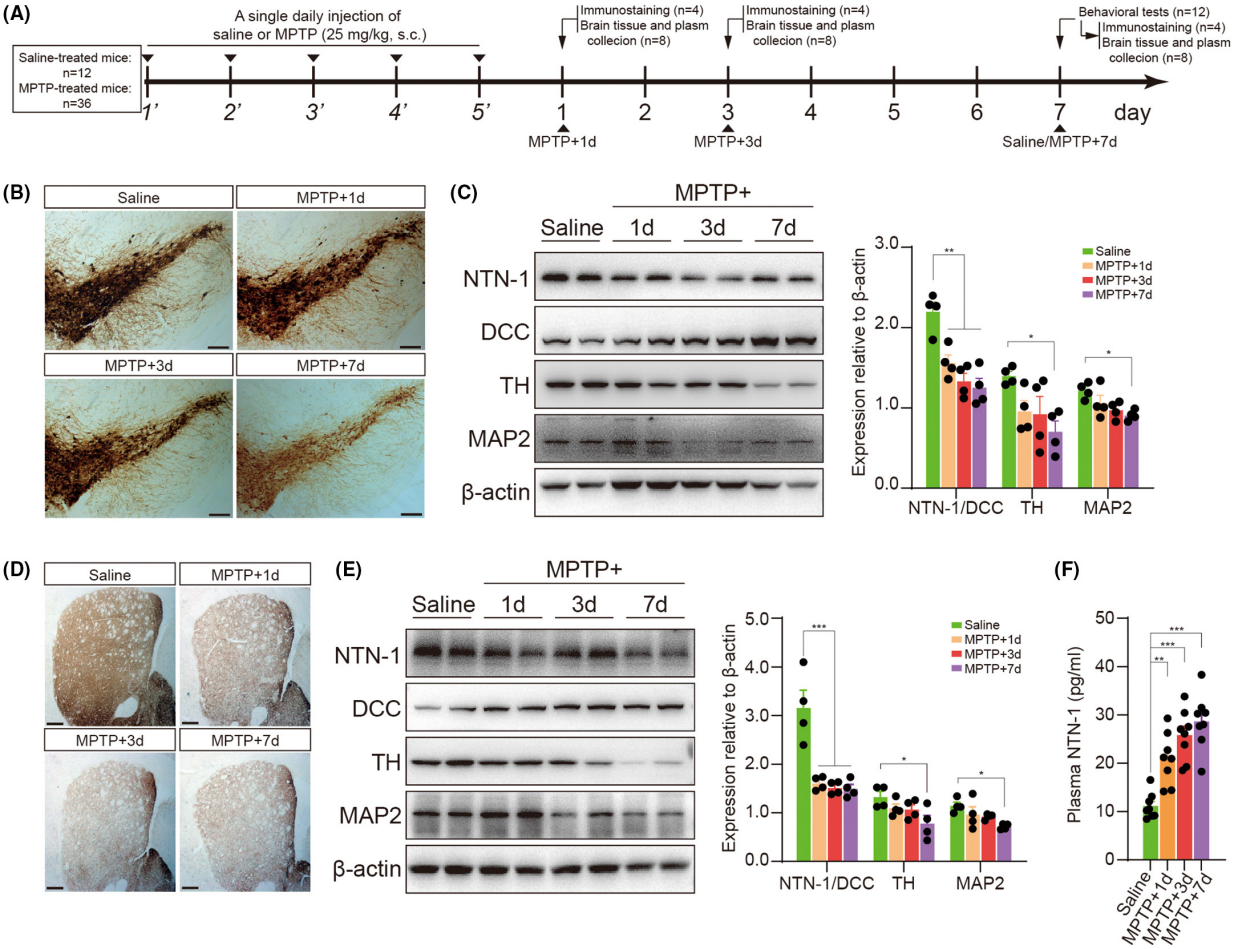
Association of NTN‐1/DCC imbalance with dopamine neurodegeneration in the subacute MPTP mouse model. (A) Experimental design in a subacute MPTP mouse model. The mice received MPTP injections every 24 h for five consecutive days. Blood samples and brains were collected from mice on days 1 (MPTP + 1d), 3 (MPTP + 3d), and 7 (MPTP + 7d) after the last injection. (B) Representative images of TH+ neurons in the SNpc on days 1, 3, and 7 after the last injection. Scale bar, 200 μm. (C) Western blotting Analysis of NTN‐1, DCC, TH, and MAP2 in the midbrain on days 1, 3, and 7 after the last injection. Data were presented as mean ± SEM and analyzed by one‐way ANOVA followed by Tukey post hoc test; *n* = 4. **p < 0.01, *p < 0.05 versus saline group. (D) Representative images of TH+ fibers in the striatum on days 1, 3, and 7 after the last injection. Scale bar, 100 μm. (E) Western blotting analysis of NTN‐1, DCC, TH, and MAP2 in the striatum on days 1, 3, and 7 after the last injection. Data were presented as mean ± SEM and analyzed by one‐way ANOVA followed by Tukey post hoc test; *n* = 4. ***p < 0.001, *p < 0.05 versus saline group. (F) Plasma NTN‐1 levels on days 1, 3, and 7 after the last injection. Data were presented as mean ± SEM and analyzed by one‐way ANOVA followed by Tukey posthoc test; n = 8. ***p < 0.001, **p < 0.01, *p < 0.05 versus saline group. IHC: immunohistochemistry; WB, western botting; MPTP: 1‐Methyl‐4‐phenyl‐1,2,3,6‐tetrahydropyridine; NTN‐1, netrin‐1; DCC: deleted in colorectal carcinoma; TH: tyrosine hydroxylase; MAP2, microtubule‐associated protein 2.

**FIGURE 2:**
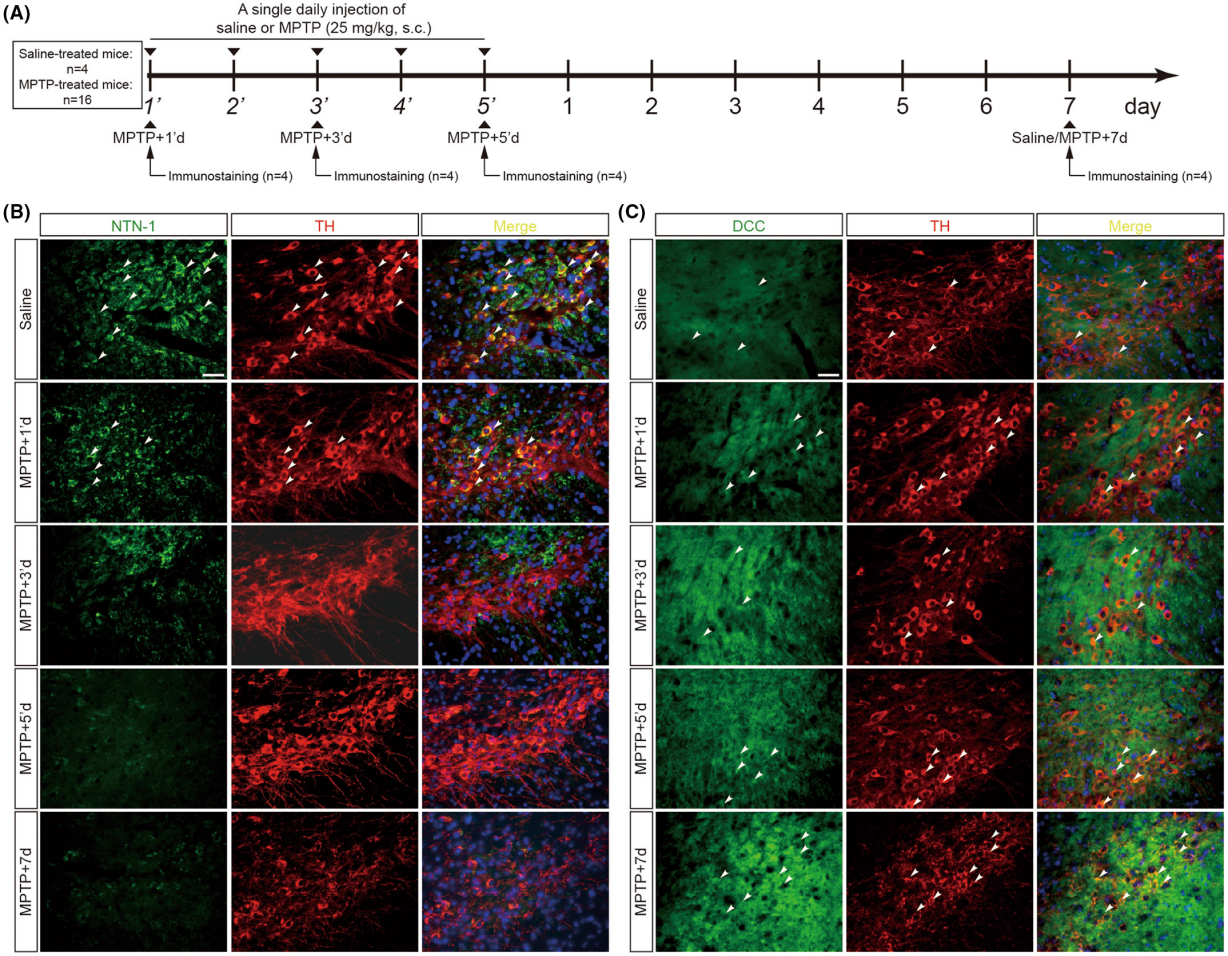
Earlier alterations of NTN‐1 and DCC in nigral dopaminergic neurons after MPTP intoxication. (A) Experimental design in a subacute MPTP mouse model. Brains were collected from mice at 6 h after 1 (MPTP‐1'd), 3 (MPTP‐3'd), or 5 (MPTP‐5'd) doses of MPTP injection and on day 7 (MPTP+7d) after the last injection. (B) Representative images of NTN‐1 (Green) in dopaminergic neurons (TH‐positive, Red) of the midbrain at 6 h after 1, 3, or 5 doses of MPTP injection and on day 7 after the last injection. Scale bar, 50 μm. (C) Representative images of DCC (Green) in dopaminergic neurons (TH‐positive, Red) of the midbrain at 6 h after 1, 3, or 5 doses of MPTP injection and on day 7 after the last injection. Scale bar, 50 μm. MPTP: 1‐Methyl‐4‐phenyl‐1,2,3,6‐tetrahydropyridine; NTN‐1, netrin‐1; DCC: deleted in colorectal carcinoma; TH: tyrosine hydroxylase.

As shown in Figure 1A, MPTP (1‐methyl‐4‐phenyl‐1,2,3,6‐tetrahydropyridine, Cat#HY‐15608, MedChemExpress, Shanghai, China; 25 mg/kg body weight, in saline, *s.c*.) was administered at 24 h intervals for five consecutive days. The same volume of saline was injected as a control. Blood samples and brains were collected from mice on Days 1 (referred to as MPTP + 1d), 3 (referred to as MPTP + 3d), or 7 (referred to as MPTP + 7d) after the last injection. We performed behavioral tests 7 days after the last injection to verify whether the model mice developed Parkinson‐like manifestations. For investigating earlier alterations of NTN‐1 and DCC, mice were sacrificed 6 h after 1 (referred to as MPTP‐1d), 3 (referred to as MPTP‐3d), or 5 (referred to as MPTP‐5d) doses of MPTP injection (Figure 2A).

#### 2.2.2 Stereotaxic LPS model of PD

As shown in Figure S2A, mice were anesthetized with pentobarbital sodium (40 mg/kg, *i.p*.), and then the midbrains of mice were bilaterally microinjected with LPS (*Escherichia coli* 0111:B4; Cat#L4516, Sigma–Aldrich, Shanghai, China; 0.5 μg LPS dissolved in 1 μL sterile saline into the brain) using the following coordinates relative to the bregma: A/P = −3.0 mm, R/L = ±1.3 mm, and D/V = −4.5 mm. The respective controls were injected with equivalent volumes of sterile saline. Brain samples were collected on day 7 after LPS injections for the subsequent immunohistochemistry and western blot experiments.

#### 2.2.3 Chronic MPTP/p model of PD

As shown in Figure S2D, MPTP (25 mg/kg body weight, in saline, *s.c*.) and a clearance inhibitor probenecid (Cat#HY‐B0545, MedChemExpress, Shanghai, China; 250 mg/kg body weight, in saline, *i.p*.) was administered twice a week for 5 weeks. Brain samples were collected from mice on day 7 after the last injection.

### 2.3 Behavioral assessments

#### 2.3.1 Open field test

Seven days after the last injection, mice were individually placed in an open field apparatus (50 × 50 cm) for 5 min. The total distance was measured and analyzed using the TopScan system (CleverSys Inc., Reston, USA).

#### 2.3.2 Accelerating rotarod test

Mice were trained for 3 days on the rotarod at a constant speed of 4 rpm, as previously described.^22^ Seven days after the last injection, mice were placed on the rotarod at speeds accelerating from 4 to 40 rpm in 5 min. In three successive trials, the latency to fall from the rotarod was measured, and mean latency values were calculated.

#### 2.3.3 Pole test

As previously described,^23^ the time needed for the mouse to turn completely head downward (T‐turn) and the total time until the mouse reached the floor with its four paws (T‐total) were recorded. T‐turn and T‐total were the best performances for each session of the trial.

### 2.4 Immunohistochemistry

As previously reported,^24^ brains were dissected, maintained in 4% paraformaldehyde overnight, cryopreserved in 30% sucrose in PBS, and stored at −70°C until used. Twenty‐micrometer coronal sections were cut and incubated with anti‐TH antibodies (Cat#T2928, 1:4000, Sigma–Aldrich, Shanghai, China) overnight at 4°C. These sections were incubated with HRP‐conjugated secondary antibodies (Cat#31430, 1:10,000, ThermoFisher, Shanghai, China) at room temperature for 1 h. Finally, according to the manufacturer's instructions, immunoreactivity was visualized by incubation in DAB (Cat#KGP1045‐100, KeyGEN BioTECH, Nanjing, Jiangsu, China).

### 2.5 Cell culture and treatment

Human SH‐SY5Y neuroblastoma cells were cultured as previously described.^24^ Cells were incubated with different MPP+ concentrations (Cat#D048, Sigma–Aldrich, Shanghai, China; 0, 20, 50, 100, 200, or 500 μM) for 24 h or with 100 μM MPP+ for a variable time period up to 72 h. To evaluate the protective effects of NTN‐1, cells were treated with recombinant human NTN‐1 (Cat#6419‐N1/CF, R&D Systems, Shanghai, China; 0, 0.001, 0.01, 0.1, or 1 μg/mL) 24 h before MPP+ stimulation (Pretreatment), with MPP+ stimulation (Cotreatment), or 24 h after MPP+ stimulation (Posttreatment). The MTT assay was used to evaluate the cell viability.

### 2.6 Western blot analysis

Brain samples or cells were lysed as previously described.^25^ The proteins were denatured in aSn DS sample buffer, separated by 10% SDS‐PAGE, and transferred to PVDF membranes. Blots were blocked with 5% skimmed milk in Tris‐buffered saline with 0.1% Tween 20 (TBST) for 1 h and then washed three times with TBST buffer for 10 min each. The blots were then incubated with the following primary antibodies: NTN‐1 (Cat#ab126729, 1:1000, Abcam, Cambridge, UK), DCC (Cat#ab273570, 1:1000, Abcam, Cambridge, UK), TH (1:1000), MAP2 (Cat#4542, 1:2000, Cell Signaling Technology, Danvers, MA, USA), p‐FAK (Cat#3283, 1:2000, Cell Signaling Technology, Danvers, MA, USA), total‐FAK (Cat#3285, 1:2000, Cell Signaling Technology, Danvers, MA, USA), p‐Src (Cat#6943, 1:2000, Cell Signaling Technology, Danvers, MA, USA), total‐Src (Cat#2109, 1:2000, Cell Signaling Technology, Danvers, MA, USA), Bax (Cat#2772, 1:2000, Cell Signaling Technology, Danvers, MA, USA), Bcl‐2 (Cat#3498, 1:2000, Cell Signaling Technology, Danvers, MA, USA), caspase‐3 (Cat#14220, 1:2000, Cell Signaling Technology, Danvers, MA, USA), or β‐actin (Cat#YFMA0052, 1:10,000, Yifeixue Biotech, Nanjing, Jiangsu, China) overnight at 4°C. The membranes were washed four times with TBST for 10 min, then incubated with the secondary antibody. Finally, the membrane was observed and analyzed with Tanon 5200 (Tanon Science and Technology Co. Ltd, Shanghai, China).

### 2.7 Patient recruitment

The study was approved by the Ethics Review Committee of the Affiliated Wuxi No. 2 People's Hospital of Nanjing Medical University (No. 2020‐Y‐2). One hundred participants, including 50 patients with PD (referred to as PD) and 50 healthy controls (referred to as HC), were recruited at the Department of Neurology of the Affiliated Wuxi No. 2 People's Hospital of Nanjing Medical University. An experienced neurologist made a definitive clinical diagnosis of PD according to the Movement Disorder Society Clinical Diagnostic Criteria for PD. Participants in PDs did not have the following^1^: familial PD,^2^ secondary PD (drug‐induced and vascular PD),^3^ atypical parkinsonism syndrome (multiple system atrophy, progressive supranuclear palsy, or dementia of Lewy body),^4^ PD with dementia, and^5^ infarction, type 2 diabetes, hemorrhage, tumors, or trauma. Meanwhile, age‐matched HCs were enrolled and recruited without a history of neurologic or psychiatric diseases, alcohol or substance abuse, psychiatric illness, or head injury. Clinical information was collected, including initial presentation, sex, age, BMI, and disease duration. Patients' disease severity and functional status were assessed by the modified Hoehn and Yahr staging scale (H‐Y stage) and Unified Parkinson's Disease Rating Scale (UPDRS).

### 2.8 Blood sampling and assaying of plasma NTN‐1

Blood was collected from all participants using a 10‐mL K2‐EDTA tube (Labtub, Shanghai, China), then gently inverted 10 times and left at room temperature for 1 h. The centrifugation was conducted at 3000 r/min for 15 min. Subsequently, 0.5 mL of plasma (supernatant) was transferred to a fresh 1.5 mL Eppendorf tube and was frozen at −80°C. NTN‐1 concentrations in human plasma were determined with an enzyme‐linked immunosorbent assay (ELISA) detection kit (CSB‐E11899h, Cusabio Biotech, Wuhan, China) following the manufacturers' instructions. NTN‐1 concentrations in mice plasma were determined with an ELISA detection kit (CSB‐EL016127MO, Cusabio Biotech, Wuhan, China).

### 2.9 Statistical analysis

The continuous data were expressed as the mean ± SEM or as the median (first quartile, third quartile), depending on the distribution. Statistical analyses were performed by GraphPad Prism software 9.0 (GraphPad Software, San Diego, CA, USA). Normality was evaluated by the Shapiro–Wilk test. Normally distributed data were analyzed by two‐sided *t* test or one‐way analysis of variance (ANOVA) followed by Tukey posthoc test, and nonparametric data were analyzed by Mann–Whitney test. The enumeration data were presented as case numbers or percentages, and two groups were compared using a Chi‐squared test. The correlation between plasma NTN‐1 levels and clinical factors such as the H‐Y stage and UPDRS was analyzed with Pearson correlation. *p* < 0.05 was considered to be statistically significant.

## 3 RESULTS

### 3.1 An imbalance of NTN‐1 and DCC is associated with dopamine neurodegeneration in mice

After five MPTP injections in the subacute model, mice were analyzed on days 1, 3, and 7 after the last MPTP injection (Figure 1A). As expected, MPTP‐treated mice showed reduced total distance traveled (*p* = 0.003, two‐sided *t* test; Figure S1A) in the open field, exhibited a longer latency period in the rotarod test (*p* < 0.001, Mann–Whitney test; Figure S1B), and displayed increased times in the pole test (T‐turn: *p* < 0.001, two‐sided *t* test; Figure S1C; T‐total: *p* = 0.008, two‐sided *t* test; Figure S1D). Meanwhile, there was a robust decline in TH‐positive cells in the substantia nigra pars compacta (SNpc) following MPTP (Figure 1B). MPTP also reduced protein expression of TH (*F*(3, 12) = 3.893, *p* = 0.037, one‐way ANOVA, Figure 1C) and MAP2 (*F*(3, 12)=4.412, *p* = 0.026, one‐way ANOVA, Figure 1C) in the midbrain. The loss of dopaminergic neurons was accompanied by a gradual decline in NTN‐1 levels and a gradual rise in DCC levels (Figure 1C). During the week following injection, the ratio of NTN‐1 to DCC in the midbrain decreased dramatically (*F*(3, 12) = 15.63, *p* < 0.001, one‐way ANOVA, Figure 1C). In the striatum, a similar phenomenon was observed. As a result of MPTP administration, the density of TH‐positive striatal fibers decreased (Figure 1D), accompanied by decreased protein levels of TH (*F*(3, 12) = 3.565, *p* = 0.047, one‐way ANOVA, Figure 1 E) and MAP2 (*F*(3, 12) = 4.050, *p* = 0.033, one‐way ANOVA, Figure 1 E). Likewise, the ratio of NTN‐1 to DCC in the striatum was dramatically reduced (*F*(3, 12) = 17.75, *p* < 0.001, one‐way ANOVA, Figure 1E). Interestingly, the levels of NTN‐1 in plasma were gradually increased (Saline: 11.14 ± 0.95 pg/mL; MPTP+1d: 20.93 ± 1.87 pg/mL; MPTP + 3d: 25.81 ± 1.84 pg/mL; MPTP + 7d: 28.66 ± 2.03 pg/mL; *F*(3, 28) = 19.89, *p* < 0.001, one‐way ANOVA, Figure 1F) after subacute MPTP administration. The gradual decline in the ratio NTN‐1 to DCC (*p* < 0.001, two‐sided *t*‐text, Figure S2C), as well as the loss of dopaminergic neurons (Figure S2B) and reduction in TH expression (*p* < 0.001, two‐sided *t*‐text, Figure S2C), were also observed in subacute models induced by inflammation‐driven stereotaxic LPS.

To mimic the slow development of human pathology, we then repeated injections of MPTP 10 times for 5 weeks to create the chronic model (Figure S2D). The MPTP/p chronic model showed a reduction in TH‐positive cells in the SNpc (Figure S2E) and a decrease in TH protein levels in the midbrain (*p* = 0.011, two‐sided *t* test, Figure S2F). There was also a dramatic decline in NTN‐1 levels, a significant increase in DCC levels, and a decreased ratio of NTN‐1 to DCC levels in the SNpc (*p* = 0.023, two‐sided *t* test, Figure S1F). These mouse models suggest that the imbalance of the NTN‐1/DCC pathway is a common feature of dopaminergic neurodegeneration.

### 3.2 Alteration of NTN‐1 and DCC precedes the death of dopaminergic neurons in the subacute MPTP model

Although the expression of NTN‐1 and DCC in substantia nigra has been observed during the late phase of the murine MPTP subacute model, earlier alteration of nigral NTN‐1 and DCC in MPTP‐treated mice is uncertain. We performed an in situ double‐immunofluorescent assay for NTN‐1 or DCC with a marker for labeling dopaminergic neurons TH over a time course of the MPTP subacute model (Figure 2A). In the saline‐treated group, NTN‐1 is found predominantly in the cytoplasm of dopaminergic neurons in the substantia nigra (Figure 2B). NTN‐1 expression was significantly reduced as early as 6 h following the first MPTP injection, and this reduction persisted during successive MPTP insults (Figure 2B). Then, we sought to identify DCC protein localization in the nigrostriatal pathway. The expression of DCC protein was weak in TH^+^ neurons of the SNpc in the sham group and gradually increased over time during MPTP injections. Especially, DCC protein was expressed highly in the remaining TH^+^ neurons on day 7 after the last MPTP injection (Figure 2C). The results of the double‐immunofluorescence assay indicate that the alterations of NTN‐1 and DCC occurred first, before the loss of dopaminergic neurons.

### 3.3 Dose‐ and time‐dependent alterations of NTN‐1 and DCC after MPP+ stimulation in SH‐SY5Y cells

**FIGURE 3:**
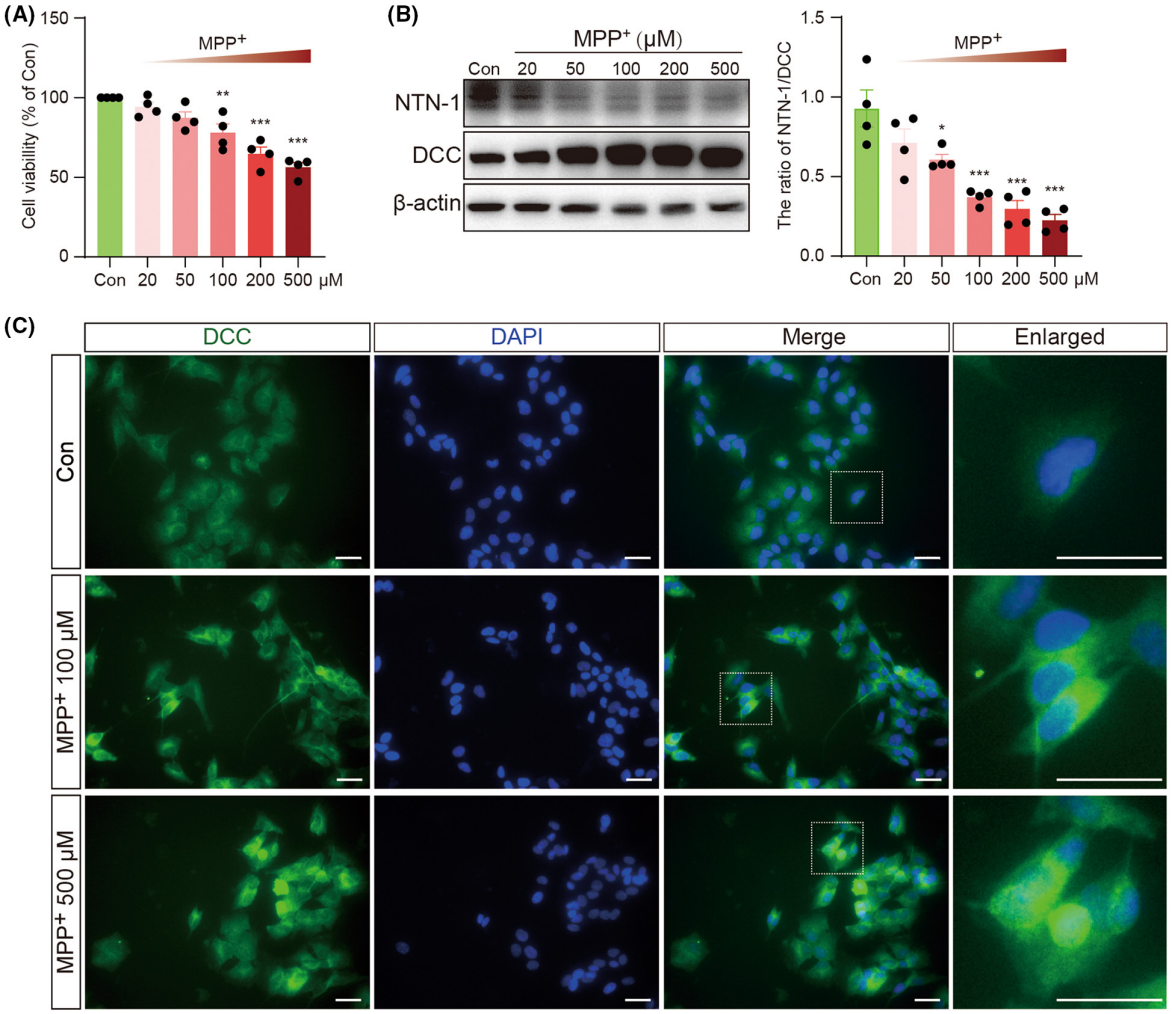
Dose‐dependent alterations of NTN‐1 and DCC after MPP+ stimulation in SH‐SY5Y cells. (A) Cell viability at 24 h after MPP+ stimulation was measured using the MTT assay. Data were presented as mean ± SEM for four independent experiments with three multiple holes in each group and analyzed by one‐way ANOVA followed by Tukey post hoc test. ***p < 0.001, **p < 0.01 versus Con group. (B) Western blotting analysis of NTN‐1 and DCC at 24 h after different doses of MPP+ in SH‐SY5Y cells. Data were presented as mean ± SEM for four independent experiments in each group and analyzed by one‐way ANOVA followed by Tukey posthoc test. ***p < 0.001, *p < 0.05 versus Con group. (C) Representative images of DCC (Green) in SH‐SY5Y cells at 24 h after 100 μM or 500 μM MPP+ stimulation. Scale bar, 100 μm. MPP+, 1‐Methyl‐4‐phenylpyridinium iodide; DCC, deleted in colorectal carcinoma; Con, control.

To further investigate the relationship between the NTN‐1/DCC pathway and cell death, human SH‐SY5Y neuroblastoma cells were exposed to different concentrations of MPP+ for 48 h. MPP+ caused a dose‐dependent loss of cell viability as judged by the MTT assay (*F*(5, 18) = 21.90, *p* < 0.001, one‐way ANOVA, Figure 3A). The cell viability decreased by 5.70% at exposure to 20 μM MPP+, 12.68% at 50 μM, 21.96% at 100 μM, 35.23% at 200 μM, and 43.72% at 500 μM. In the MPP+‐treated cells, NTN‐1 protein levels were decreased while DCC levels were elevated, resulting in a dose‐dependently decreased ratio of NTN‐1/DCC (*F*(5, 18) = 16.37, *p* < 0.001, one‐way ANOVA, Figure 3B). Immunofluorescence results further confirmed the increased expression of DCC in MPP+‐treated cells (Figure 3C).

**FIGURE 4:**
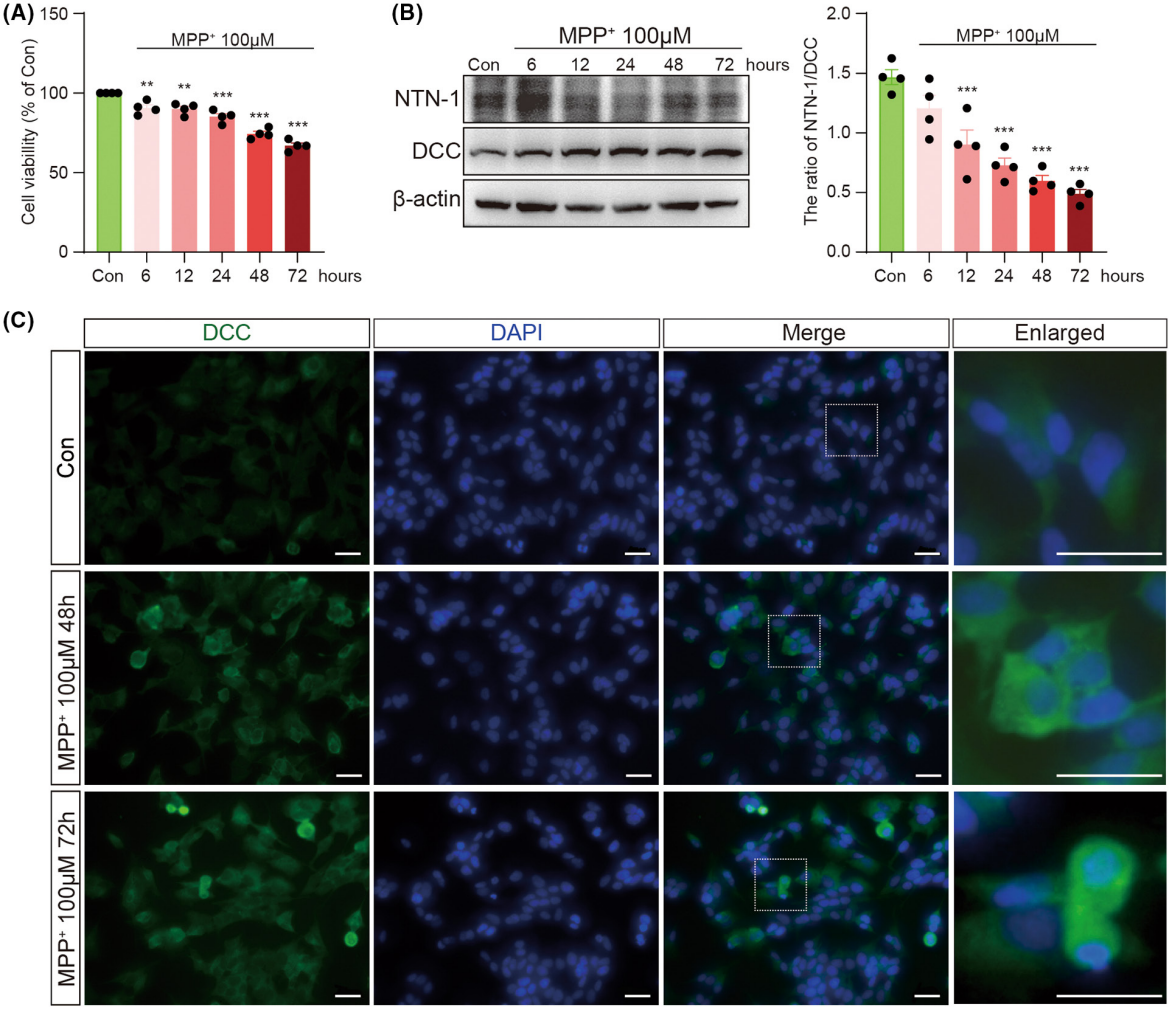
Time‐dependent alterations of NTN‐1 and DCC after MPP+ stimulation in SH‐SY5Y cells. (A) Cell viability at different times after 100 μM MPP+ stimulation was measured using the MTT assay. Data were presented as mean ± SEM for four independent experiments with three multiple holes in each group and analyzed by one‐way ANOVA followed by Tukey post hoc test. ***p < 0.001, **p < 0.01 versus Con group. (B) Western blotting analysis of NTN‐1 and DCC at different times after 100 μM MPP+ stimulation in SH‐SY5Y cells. Data were presented as mean ± SEM for four independent experiments in each group and analyzed by one‐way ANOVA followed by Tukey post hoc test. ***p < 0.001 versus Con group. (C) Representative images of DCC (Green) in SH‐SY5Y cells at 48 and 72 h after 100 μM MPP+ stimulation. Scale bar, 100 μm. Con, control; DCC, deleted in colorectal carcinoma; MPP+, 1‐Methyl‐4‐phenylpyridinium iodide.

Next, SH‐SY5Y cells were exposed to 100 μM of MPP+, and the MTT test was performed at different time points. MPP+ caused a time‐dependent loss of cell viability (*F*(5, 18) = 51.05, *p* < 0.001, one‐way ANOVA, Figure 4A). The cell viability decreased by 9.38% at 6 h, 10.04% at 12 h, 14.85% at 24 h, 25.59% at 48 h, and 33.04% at 72 h. There was a time‐dependent decrease in the ratio of NTN‐1/DCC (*F*(5, 18) = 22.15, *p* < 0.001, one‐way ANOVA, Figure 4B). Immunofluorescence results confirmed the increased expression of DCC at 48 h and 72 after 100 μM of MPP+ treatment (Figure 4C).

### 3.4 Dose‐ and time‐dependent alterations of NTN‐1/DCC signaling after MPP+ stimulation in SH‐SY5Y cells

**FIGURE 5:**
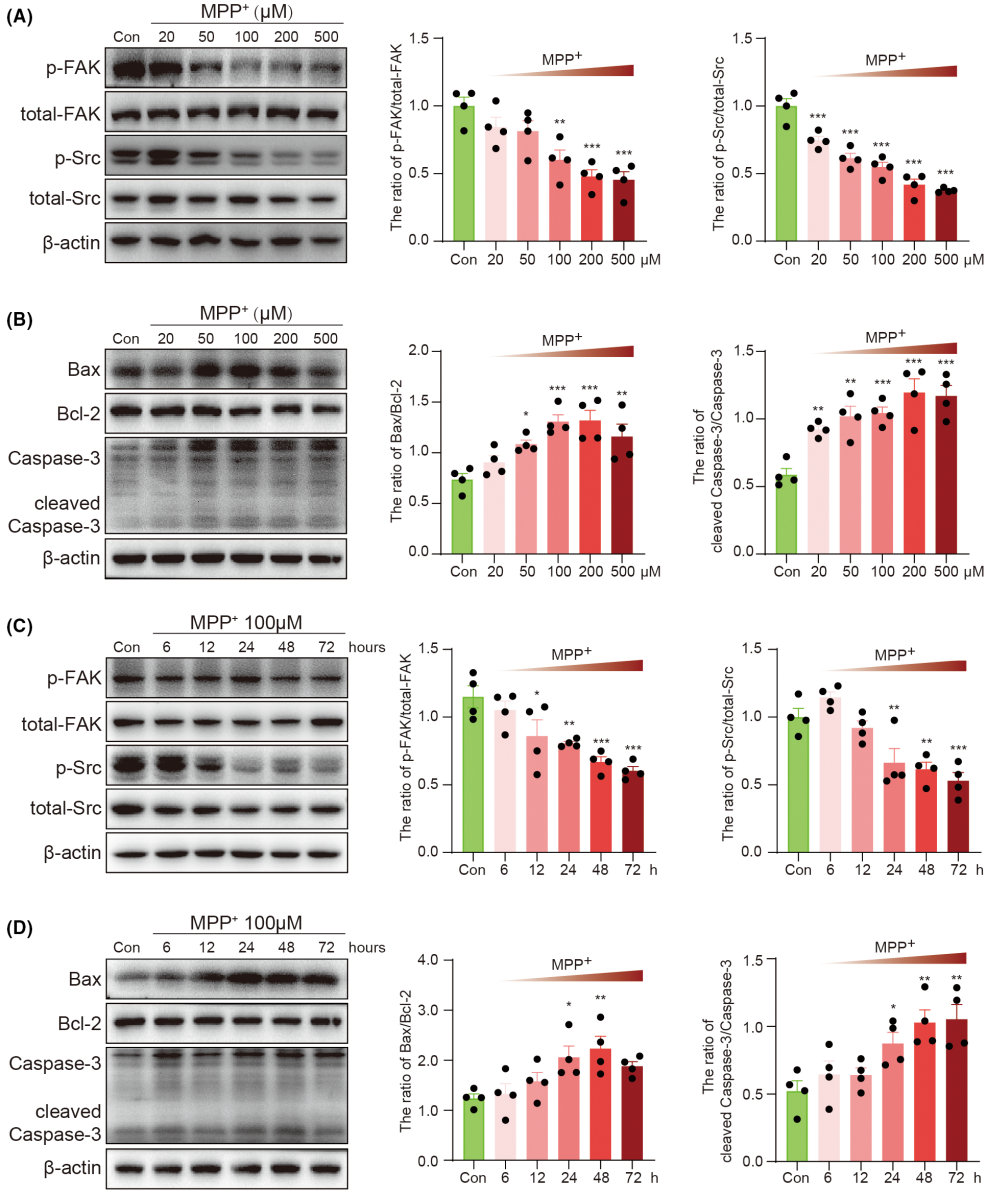
Dose‐ and time‐dependent alterations of NTN‐1‐DCC signaling after MPP+ stimulation in SH‐SY5Y cells. (A) Western blotting analysis of p‐PAK and p‐Src at 24 h after different doses of MPP+ stimulation in SH‐SY5Y cells. Data were presented as mean ± SEM for four independent experiments in each group and analyzed by one‐way ANOVA followed by Tukey post hoc test. ***p < 0.001, **p < 0.01 versus Con group. (B) Western blotting analysis of Bax, Bcl‐2, cleaved caspase‐3, and caspase‐3 at 24 h after different doses of MPP+ stimulation in SH‐SY5Y cells. Data were presented as mean ± SEM for four independent experiments in each group and analyzed by one‐way ANOVA followed by Tukey post hoc test. ***p < 0.001, **p < 0.01, *p < 0.05 versus Con group. (C) Western blotting analysis of p‐PAK and p‐Src at different times after 100 μM MPP+ stimulation in SH‐SY5Y cells. Data were presented as mean ± SEM for four independent experiments in each group and analyzed by one‐way ANOVA followed by Tukey post hoc test. **p < 0.001, **p < 0.01, *p < 0.05 versus Con group. (D) Western blotting analysis of Bax, Bcl‐2, cleaved caspase‐3, and caspase‐3 at different times after 100 μM MPP+ stimulation in SH‐SY5Y cells. Data were presented as mean ± SEM for four independent experiments in each group and analyzed by one‐way ANOVA followed by Tukey post hoc test. ***p < 0.001, **p < 0.01, *p < 0.05 versus Con group. Con, control; DCC, deleted in colorectal carcinoma; MPP+: 1‐methyl‐4‐phenylpyridinium iodide; NTN‐1, netrin‐1.

As a dependence receptor, DCC has different functions in the presence or absence of NTN‐1. With NTN‐1, DCC mediates axon growth and chemoattractive function, which requires the activation of focal adhesion kinase (FAK) – Src signaling pathway. Without NTN‐1, DCC triggers apoptosis through an apoptosome‐independent activation of caspase‐3. To confirm the involvement of FAK and Src in DCC signaling, we observed that MPP+‐stimulated phosphorylation of FAK and Src. MPP+ inhibited phosphorylated FAK (*F*(5, 18) = 10.82, *p* < 0.001, one‐way ANOVA, Figure 5A) and phosphorylated Src (*F*(5, 18) = 38.78, *p* < 0.001, one‐way ANOVA, Figure 5A) in a dosage‐dependent manner. A significant increase was observed in the ratio of cleaved caspase‐3 to caspase‐3 (*F*(5, 18) = 11.13, *p* < 0.001, one‐way ANOVA, Figure 5B) and the ratio of Bax to Bcl‐2 (*F*(5, 18)=7.87, *p* < 0.001, one‐way ANOVA, Figure 5B) in MPP + ‐treated cells. Meanwhile, MPP+ also inhibited phosphorylated FAK (*F*(5, 18) = 9.68, *p* < 0.001, one‐way ANOVA, Figure 5C) and phosphorylated Src (*F*(5, 18) = 13.91 *p* < 0.001, one‐way ANOVA, Figure 5C) in a time‐dependent manner. As expected, a significant increase was observed in the ratio of cleaved caspase‐3 to caspase‐3 (*F*(5, 18) = 6.45, *p* = 0.001, one‐way ANOVA, Figure 5D) and the ratio of Bax to Bcl‐2 (*F*(5, 18) = 4.71, *p* = 0.006, one‐way ANOVA, Figure 5D) in MPP+‐treated cells. However, exogenous NTN‐1 administration did not alleviate MPP+‐induced loss of cell viability in pretreatment (Figure S3A), cotreatment (Figure S3B), or posttreatment (Figure S3C).

### 3.5 Idiopathic PD patients have lower plasma levels of NTN‐1

**FIGURE 6:**
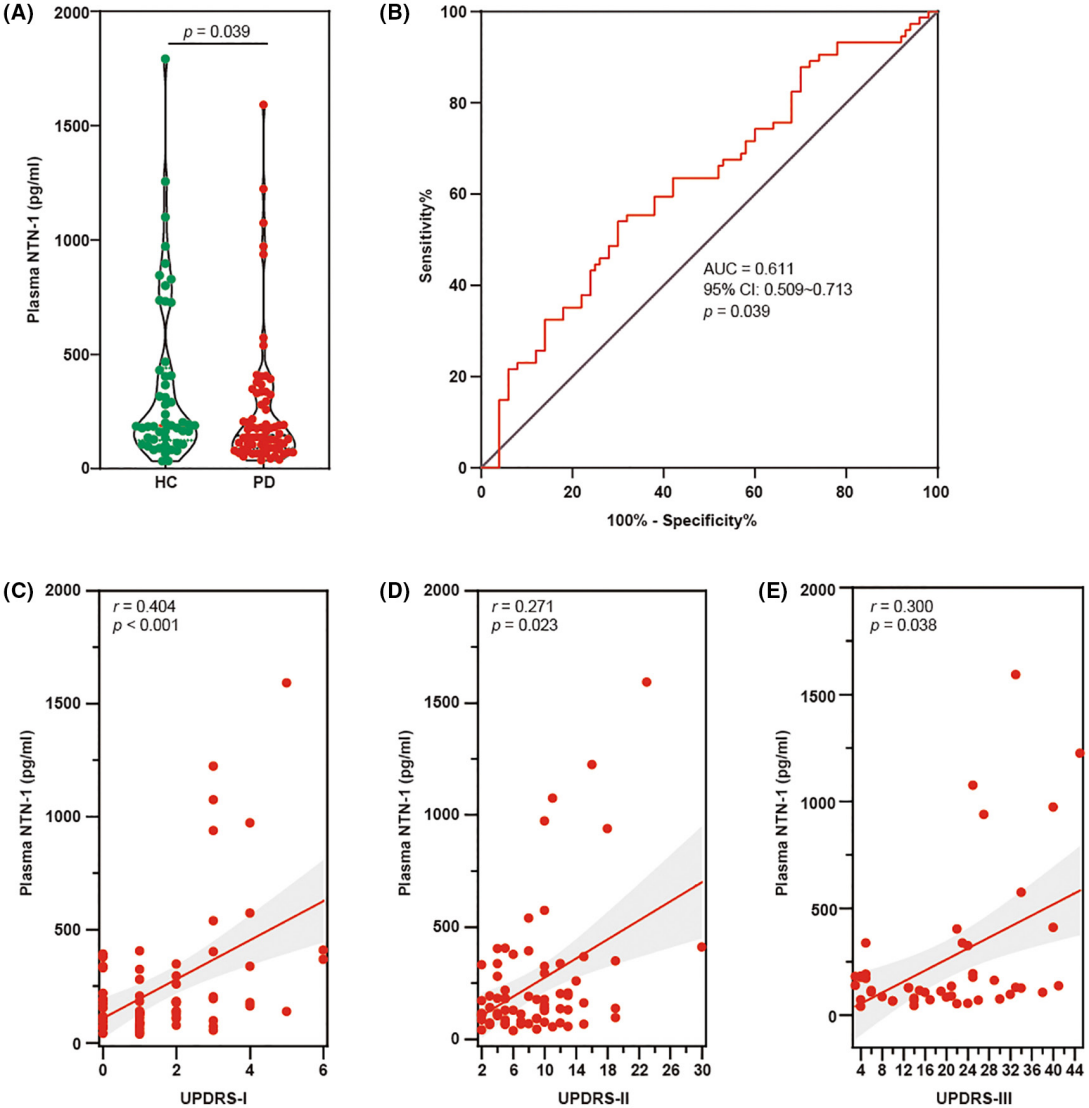
Plasma NTN‐1 level and its correlation with UPDRS score in PD patients. (A) Scatter diagram of plasma NTN‐1 levels between the HC and PD groups. Data were presented as dot plots in each group and analyzed by Mann–Whitney test. (B) ROC curves of plasma NTN‐1 levels for distinguishing PD patients from HC. The whiskers in the box plots indicate the values from minimum to maximum, and the center line indicates the median. (C) Relationship between plasma NTN‐1 levels and UPDRS I among PD patients. (D) Relationship between plasma NTN‐1 levels and UPDRS II among PD patients. (E) Relationship between plasma NTN‐1 levels and UPDRS III among PD patients. AUC, area under the ROC curve; HC, healthy control; NTN‐1, netrin‐1; PD, Parkinson's disease; UPDRS, Unified Parkinson disease rating scale.

We then conducted correlation studies between plasma NTN‐1 and parkinsonian symptoms to understand how this pathway contributes to PD. The demographic data are shown in Table 1. Sex, BMI, and age in study did not significantly differ between idiopathic PD and aged‐matched HC. The median concentration of NTN‐1 in patients with idiopathic PD (133.6 (77.62–233.1) pg/mL) was significantly lower (*p* = 0.039, Mann–Whitney test, Figure 6A) than that in patients with HC (189.0 (124.4–439.8) pg/mL). The diagnostic utility of plasma NTN‐1 levels was further assessed by ROC analyses between idiopathic PD and HC. The area under the curve was 0.611 (95% confidence intervals 0.509–0.713, *p* = 0.039, Figure 6B). A cutoff value for plasma NTN‐1 of 152.7 pg/mL was selected, yielding a sensitivity of 51.43% (CI: 39.95%–62.75%) and a specificity of 70.00% (CI: 56.25%–80.90%). Interestingly, NTN‐1 levels in plasma showed a positive correlation with UPDRS part I (*r* = 0.404, *p* < 0.001, Figure 6C), II (*r* = 0.271, *p* = 0.023, Figure 6D), and III (*r* = 0.300, *p* = 0.038, Figure 6E) in idiopathic PD. NTN‐1 levels did not correlate with age (Figure S4A), BMI (Figure S4B), course of disease (Figure S4C), and H‐Y stage (Figure S4D). It is worth noting that if the data of only one patient in Stage 4 were removed, the *p* value of correlation between NTN‐1 and H‐Y stage would reach 0.058 (Figure S4E).

**TABLE 1:** Clinical characteristics of the patients with PD and healthy controls.

	HC	PD	*p* Value
*n*	50	70	
Age (years)	66.88 ± 1.51	69.90 ± 1.04	*p* = 0.091
Gender (male, %)	62.0	62.9	*p* = 0.924
BMI	23.21 ± 0.32	23.16 ± 0.37	*p* = 0.924
Duration (years)	‐	3 (1.00–5.25)	N.A
H‐Y stage		2.00 (1.00–2.50)	N.A

## 4 DISCUSSION

Netrin‐1 and its receptor DCC are highly expressed in adult nigral dopamine neurons,^20^, ^26^ suggesting that they may be involved in the function or degeneration of these cells. Recently, Jasmin et al. found that silencing NTN‐1 in the adult substantia nigra of mice leads to the cleavage of DCC and loss of dopamine neurons.^20^ In the 6‐hyroxydopamine‐lesioned rat model and A53T‐SNCA mouse PD model, a similar therapeutic potential exists for overexpressing NTN‐1 and injecting recombinant NTN‐1 into the brain.^20^ In addition, they found a reduction in NTN‐1 expression among postmortem brain samples and gut biopsies from PD patients.^20^, ^27^ In the present study, we found that the imbalance of the NTN‐1/DCC pathway is a common feature of dopaminergic neurodegeneration and, for the first time, revealed that plasma NTN‐1 levels were significantly lower in PD patients than in aged‐matched HC.

In the present study, we found that the neurodegeneration of nigral DA neurons was always accompanied by an imbalance of NTN‐1 and DCC, as shown in MPTP and LPS PD models. Using NTN‐1 conditional knockout (KO) mice, Jasmin et al. found that deleting NTN‐1 in the SNpc caused severe motor impairment and a loss of nigral DA neurons, accompanied by increased levels of DCC receptors.^20^ Further experiments in aged DAT^cre^/DCC^fl/fl^ mice revealed a significant decrease in ventral midbrain DA neurons.^21^ These findings are in line with our speculation that the imbalance between NTN‐1 and DCC should be a common feature of DA neuron injury.

Furthermore, we found that subacute MPTP exposure resulted in earlier onset of these imbalances than a reduction of TH expression in the SNpc. In the brain, MPTP is converted to its final toxic metabolite MPP^+^, which selectively enters DA neurons via the DA transporter and then inhibits Complex I of the electron transport system in the mitochondria. Studies by Jackson‐Lewis et al. proved that a nigral DA degeneration phase began at 12 h postinjection of MPTP and persisted for 4 days.^28^ TH‐defining neurons showed a greater loss during this period than Nissl‐stained neurons, suggesting MPTP can reduce TH without necessarily killing the neurons. In the present study, the reduction of NTN‐1 and the increase of DCC in the SNpc were observed 6 h after the first injection of MPTP, indicating that MPTP can directly inhibit the NTN‐1/DCC pathway. Indeed, consistent with the results in animal models, studies in vitro conducted on SH‐SY5Y cells demonstrated that MPP+ inhibited NTN‐1 expression and increased DCC expression in both a concentration and time‐dependent manner. Further studies are needed to examine how MPP+ contributes to this imbalance.

Focal adhesion kinase and Src play a critical role in netrin signaling in axon outgrowth, migration, and axon attraction.^29^ Both FAK and Src stimulate DCC phosphorylation and are potential downstream effects of DCC. FAK has been implicated in the function of netrin through its links to other molecules, such as PLC‐γ, PI3K, Rac, Cdc42, and MAP kinases. MPP+ can reduce NTN‐1 expression, followed by reduced phosphorylation levels of FAK and Src in a concentration‐ and time‐dependent manner, which might explain why axon growth was inhibited before MPP+ depletes cellular ATP in previous studies.^30^ Generally, without NTN‐1, DCC leads to cell death independent of the mitochondrial pathway and the death receptor pathway.^31^ DCC activates caspase‐3 via a caspase‐9‐dependent manner, which cannot be blocked by Bcl‐2.^32^ However, MPP+ induces apoptotic cell death directly via a mitochondrial pathway, including enhanced caspase 3 activations.^33^ Thus, the MPP+ model may not be suitable for observing whether or not cells are damaged via DCC‐mediated dependence receptor pathways. Possibly because of this, exogenous supplementation of NTN‐1 did not prevent cell death in the MPP+‐induced cell model or neurodegeneration of the MPTP mouse model in Jasmin's study.^20^

In line with previously reported postmortem brain samples from PD patients, we found a reduction in plasm netrin‐1 levels in patients compared with aged‐matched HC. A reduction in serum NTN‐1 levels was also observed in patients with subclinical atherosclerosis,^34^ type 2 diabetes mellitus,^35^ and multiple sclerosis.^36^ Thus, NTN‐1, particularly in plasm, seems unable to identify DA neurodegeneration in a specific manner, depending on the specific type of nerve damage and stage of disease development.

In the present study, we discovered correlations between plasma NTN‐1 levels and UPDRS but not the H‐Y stage or disease course. The UPDRS is the most widely used scale to measure impairment and disability in PD.^37^ Although it has not been designed for classification on levels of severity, the different scores of the UPDRS may suggest categorical levels of disease severity at bedside use.^38^ The H‐Y stage is another widely used and accepted staging system for the severity of PD, which significantly correlates with both quality‐of‐life measures and studies of objective motor performance. Because the H‐Y stage is driven mainly by motor features and disability, the proportion of severity levels in UPDRS Parts II and III were highly correlated with the H‐Y stage, as previously reported.^39^ Although we only found the correlations between plasma NTN‐1 levels and UPDRS in the present study, we noticed that the P value of correlation between NTN‐1 and the H‐Y stage would reach 0.058 if the data of one patient in Stage 4 were excluded. A correlation exists between the number of dopaminergic neurons in the SNpc and disease severity assessed by the UPDRS score and H‐Y stage.^40^ This finding suggests that there could be a positive association between plasm NTN‐1 and neurodegeneration in PD. In addition, we found that plasma NTN‐1 was negatively correlated with disease course. Considering that the levels of PD severity are due to different rates of disease progression, such as the age of onset, cognitive impairment, or PD subtype, no correlation between plasma NTN‐1 and disease course may result from the heterogeneous composition of the disease. Also, patients with a long disease duration may have a better prognosis because of the overall burden during increasing disease duration.

Interestingly, plasma levels of NTN‐1 in PD patients were lower than in normal controls but positively correlated with symptom severity in patients with PD. Our results imply that plasma NTN‐1 could increase with the injury of dopaminergic neurons, especially in PD patients with H‐Y stages 1–3. We also observed that plasma NTN‐1 in the MPTP subacute model mice was gradually increased after MPTP administration. Recent evidence shows that serum NTN‐1 was associated with recovery after ischemic stroke.^41^ NTN‐1 can promote neuronal regeneration, reduces ischemia–reperfusion injury, and regulates blood–brain barrier integrity in ischemic lesions. It has been speculated that the increase in plasma NTN‐1 may act as a protective mechanism against neurondegeneration in PD. Thus, further investigating should be carried out to clarify the correlation between NTN‐1 and PD symptoms, including recruiting a larger sample size of all H‐Y stages, analyzing the relationship between circulating NTN‐1 levels and non‐movement symptoms, and obtaining centrally derived NTN‐1 from PD patients. It is needed to determine whether reduced plasma NTN‐1 results from PD susceptibility or is a consequence of neurodegeneration.

In conclusion, a significant decrease in plasma NTN‐1 was found for the first time in PD patients, with a close correlation to symptoms. Furthermore, as a common feature of nigral DA neuron injury in the early stages, the imbalance of NTN‐1 and DCC appears before cell death. However, more studies are needed to clarify the potential differences between central and peripheral NTN‐1. In addition, the NTN‐1/DCC signaling pathway needs to be tested in models other than MPTP neurotoxin to confirm whether it contributes to neurodegeneration in PD and whether targeting this signaling pathway can alleviate or even reverse the damage of DA neurons.

## AUTHOR CONTRIBUTIONS

Y.H., Y.L., and Y.F. performed study concepts and design. Y.H., B.H., Q.Y., Y.L., and D.X. designed and performed clinical studies. W.H., L.Z., J.Z., and N.S. designed and performed the animal experiment. W.H., J.Z., and J.G. carried out cellular experiments. Y.H., W.H., J.Z., and N.S. provided analysis and interpretation of data and statistical analysis. Y.H., N.S., Y.L., and Y.F. performed writing, reviewing, and revising the paper. All authors read and approved the final paper.

## CONFLICT OF INTEREST STATEMENT

The authors declare that they have no conflict of interest.

## Supporting information


Figure S1.

Figure S2.

Figure S3.

Figure S4.

## Data Availability

The raw data supporting the conclusions of this article will be made available by the authors, without undue reservation, to any qualified researcher.
