# Supplementary figures and images for: An imbalance of netrin‐1 and DCC during nigral degeneration in experimental models and patients with Parkinson's disease
Source: CNS Neurosci Ther. 2023 Feb 27;29(7):1817–29. doi: 10.1111/cns.14141 (PMC10324354; doi:10.1111/cns.14141)

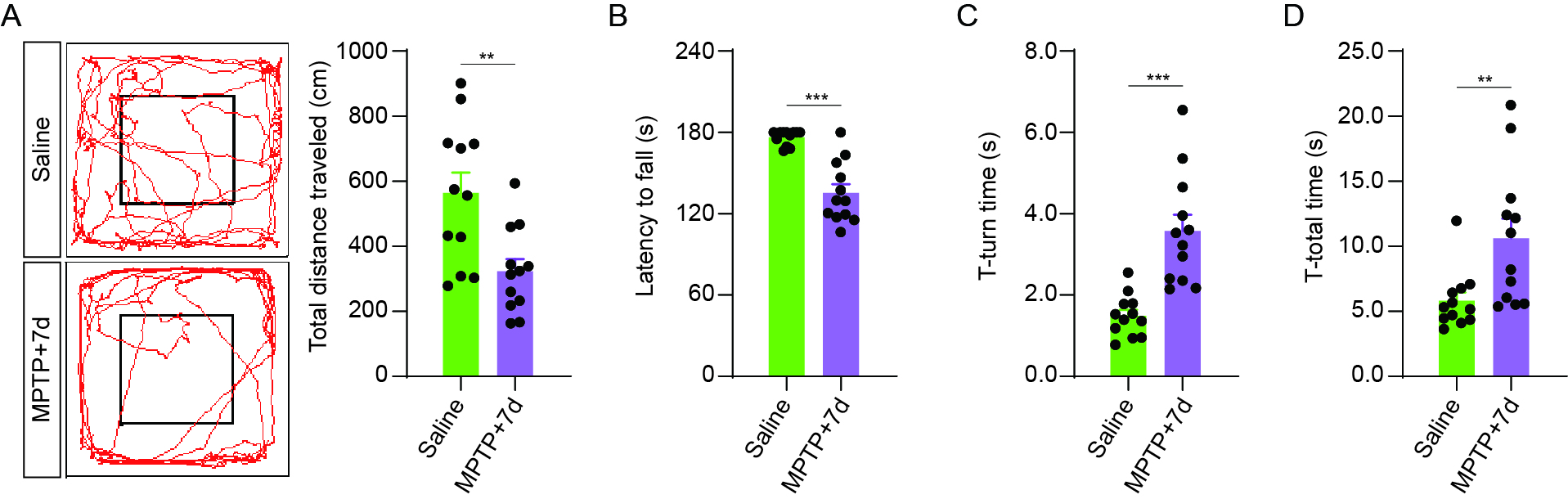

Supplement: Supplementary file 1 — Figure S1. [file CNS-29-1817-s004.jpg]

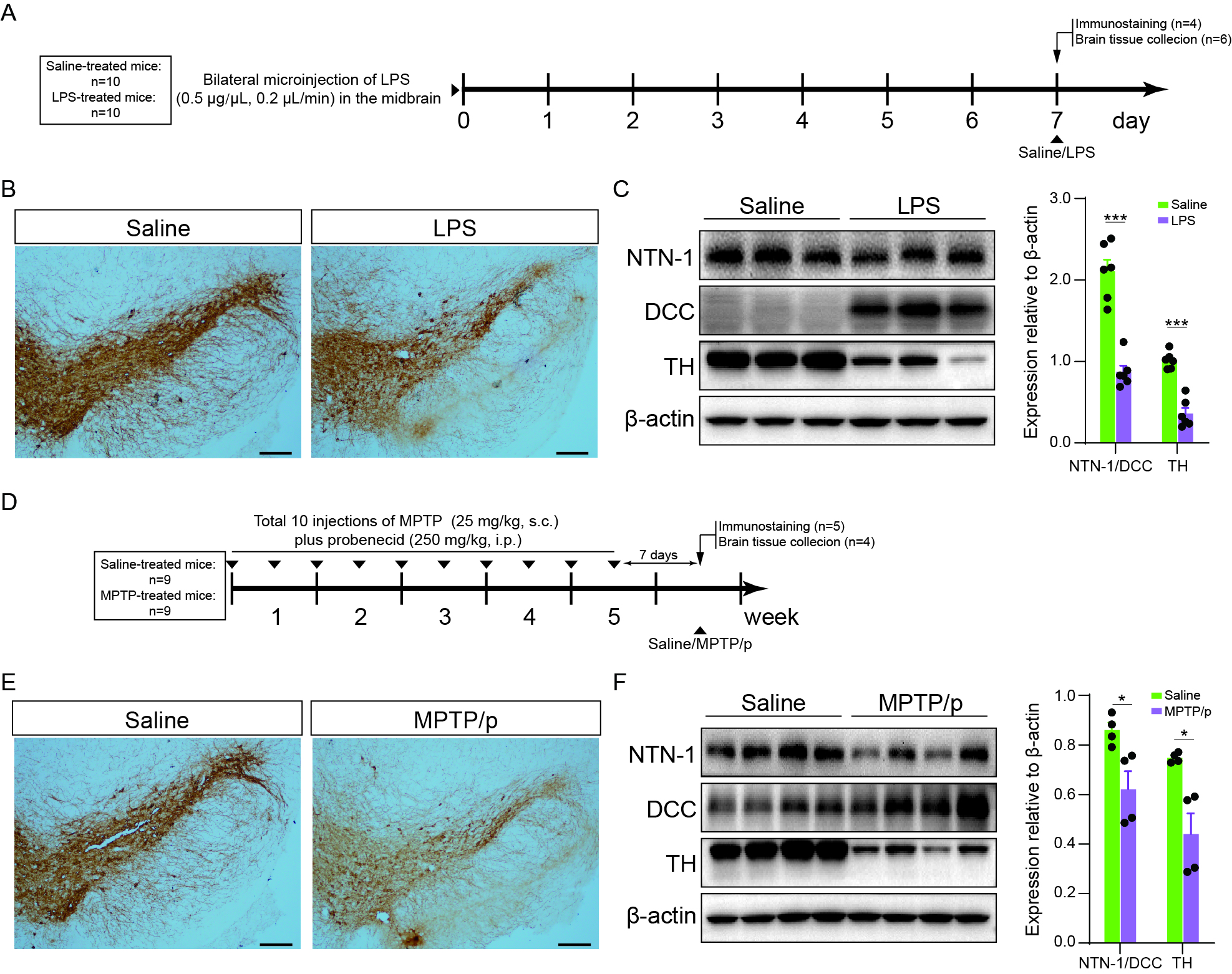

Supplement: Supplementary file 2 — Figure S2. [file CNS-29-1817-s002.jpg]

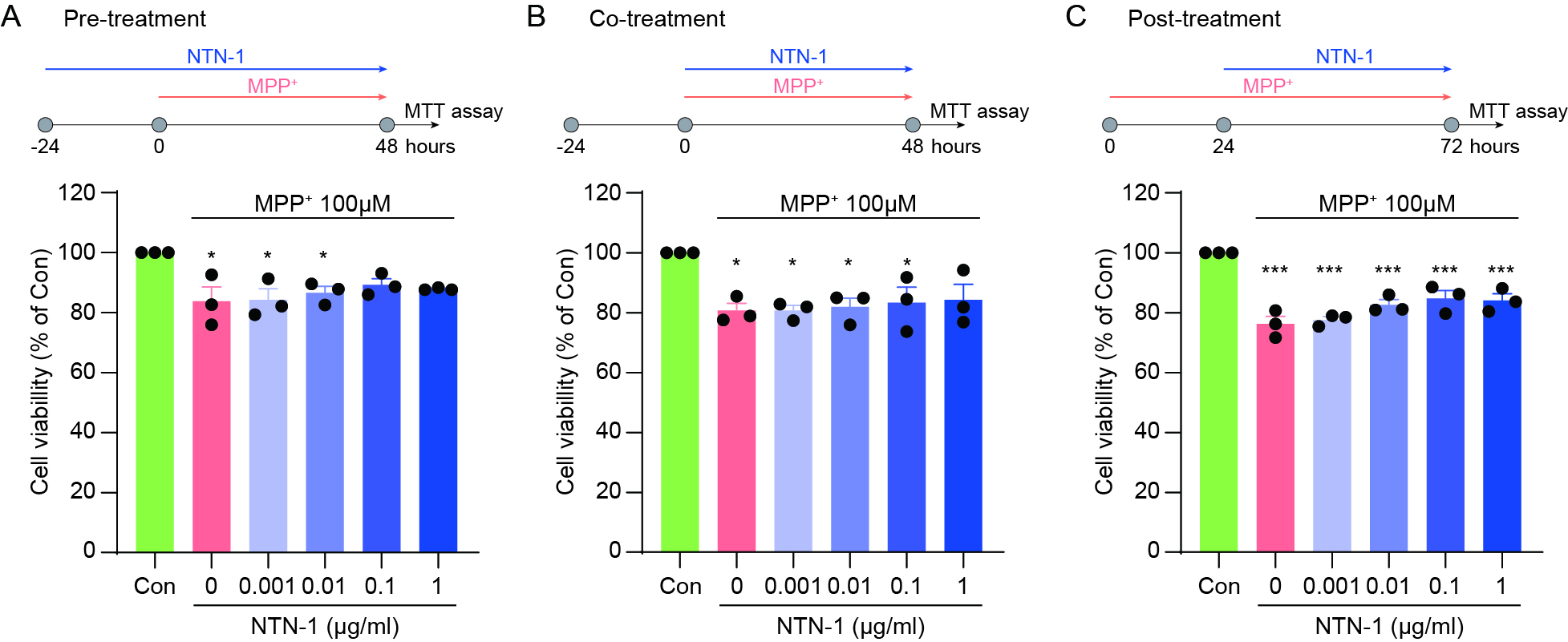

Supplement: Supplementary file 3 — Figure S3. [file CNS-29-1817-s001.jpg]

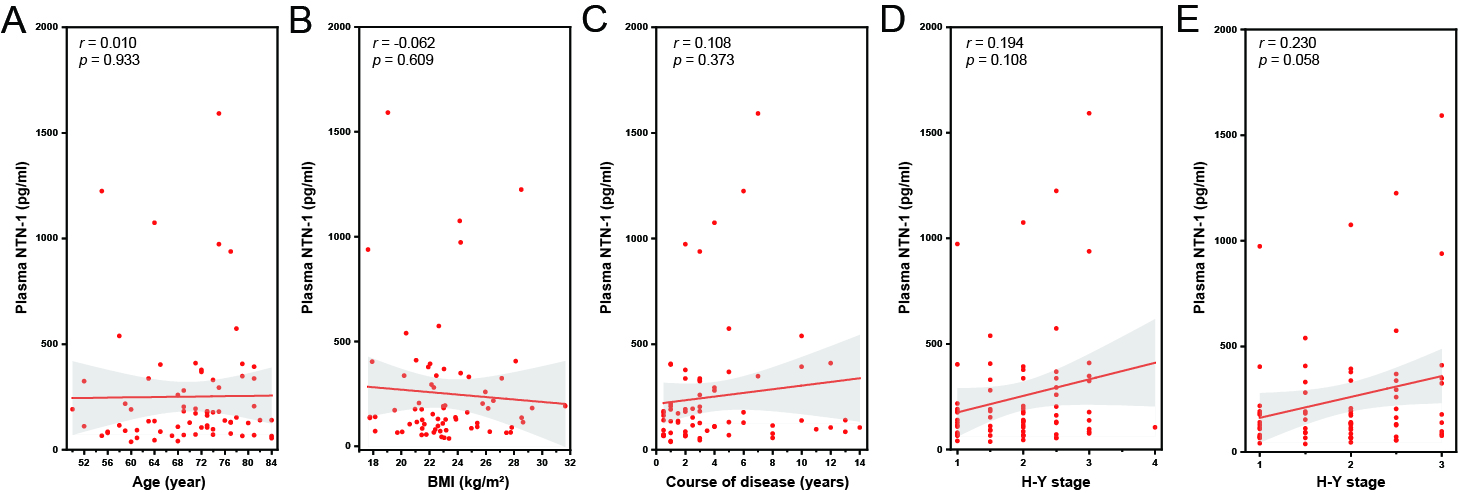

Supplement: Supplementary file 4 — Figure S4. [file CNS-29-1817-s003.jpg]
